# Microbial Symbiont-Based Detoxification of Different Phytotoxins and Synthetic Toxic Chemicals in Insect Pests and Pollinators

**DOI:** 10.3390/jox14020043

**Published:** 2024-06-04

**Authors:** Olivia Kline, Neelendra K. Joshi

**Affiliations:** Department of Entomology and Plant Pathology, University of Arkansas, Fayetteville, AR 72701, USA

**Keywords:** insecticide resistance, xenobiotics, gut microbiome, bacterial symbionts, detoxification

## Abstract

Insects are the most diverse form of life, and as such, they interact closely with humans, impacting our health, economy, and agriculture. Beneficial insect species contribute to pollination, biological control of pests, decomposition, and nutrient cycling. Pest species can cause damage to agricultural crops and vector diseases to humans and livestock. Insects are often exposed to toxic xenobiotics in the environment, both naturally occurring toxins like plant secondary metabolites and synthetic chemicals like herbicides, fungicides, and insecticides. Because of this, insects have evolved several mechanisms of resistance to toxic xenobiotics, including sequestration, behavioral avoidance, and enzymatic degradation, and in many cases had developed symbiotic relationships with microbes that can aid in this detoxification. As research progresses, the important roles of these microbes in insect health and function have become more apparent. Bacterial symbionts that degrade plant phytotoxins allow host insects to feed on otherwise chemically defended plants. They can also confer pesticide resistance to their hosts, especially in frequently treated agricultural fields. It is important to study these interactions between insects and the toxic chemicals they are exposed to in order to further the understanding of pest insect resistance and to mitigate the negative effect of pesticides on nontarget insect species like Hymenopteran pollinators.

## 1. Introduction

A recent estimate of eukaryote biodiversity predicted around 8.7 million species as the global total [1]. Of these, 5.5 million species (63.2%) have been estimated to be insects [2,3]. Insects remain the most diverse form of life, and one of the most abundant forms globally. They can be found on every continent, including Antarctica [4], and can exist in a variety of ecosystems, in marine, freshwater, and terrestrial environments. As such, they have become an important part of human life, especially through their interactions with agriculture, the economy, and human health in both positive and negative ways. The benefits they can provide include ecosystem services, such as pollination, soil aeration, nutrient cycling, and biological control of pest species [5,6,7,8].

Animal pollination, especially by bees, flies, and other insects, provides around 15–30% of the human diet, both through the direct pollination of edible crops and through the pollination of crops used for livestock feeding, such as alfalfa and clover [5,9]. Pollination increases both the yield and quality of many crops [10,11,12], which benefits the profits of farmers, amounting to billions of dollars annually in the United States alone [13,14]. Insect pollinators are particularly important for the production of vegetables, fruits, oil crops, and nuts [15]. Pollinator-dependent crops provide a large portion of nutrients to the human diet, especially vitamin C, vitamin A, and lycopene [16], and as such can help combat “hidden hunger”—in which people receive enough daily calories, but have nutrient deficiencies due to lack of access to an adequate variety of foods [9,17].

Additionally, insects can act as natural enemies of pest species, both as predators or parasitoids [18]. Generalist predators, like many ground beetles (Coleoptera: Carabidae), have been shown to reduce populations of crop pest species, including the winter moth (*Operopthera brumata*) [19,20]. Several hymenopteran parasitoids, notably the ichneumonid wasps in genus *Diadegma*, have parasitized and provided biological control of the diamondback moth (*Plutella xylostella*), which is resistant to many applied insecticides [21]. Ants can act as natural enemies, and they can additionally play a vital role as ecosystem engineers: increasing soil aeration, water filtration, and nutrient cycling [8,22]. Dung beetles (Coleoptera: Scarabaeidae) act as decomposers of waste and aid in the recycling of nutrients [7].

As pests, insects can cause severe economic damage and threats to human and livestock health. Though weeds tend to be the greatest contributor to crop yield decreases [23], insect and other arthropod pests can also contribute. In the United States, crop yield loss due to insects is estimated to reach hundreds of billions of dollars [24]. Mosquitoes can be responsible for vectoring severe diseases to humans and domesticated animals, such as malaria, dengue fever, chikungunya, and West Nile [25,26], resulting in 438,000 reported deaths globally in 2015 [27]. Other insects, like sandflies, tsetse flies, and fleas have also transmitted multiple diseases [26]. Because of the serious harm insect pests can cause to agricultural crops and to human health, managing their populations has become an important and large-scale industry.

These pest species, beneficial species, and other insects are often exposed to a wide variety of natural and synthetic toxins in the environment. Many toxins are produced by other organisms, such as phytotoxins, which are synthesized by plants in order to deter herbivorous insects from feeding on them [28]. Prey species of insects may synthesize or accumulate toxins to prevent predation, such as the monarch butterfly (*Danaus plexippus*), which sequesters cardenolide toxins from milkweed plants (*Asclepias* spp.) to make themselves more unpalatable [29]. Alternatively, some arthropod predators, like spiders, use venom to subdue or kill insect prey [30]. In agricultural and urban areas, insects are often exposed to synthetic toxins and various toxic chemicals, such as insecticides, fungicides, and other chemical pollution [31,32,33]. The interactions between insects and the toxic chemicals, both naturally occurring and synthetic, can be complicated, especially as many insects depend on microbial symbionts, such as their gut bacterial symbionts, to aid in toxin degradation [34,35,36,37]. The role of these symbionts in insect growth, health, and activity is still not fully understood, but it is apparent that microbes are capable of detoxifying many xenobiotics and conferring resistance on several insect species [37,38,39,40,41]. Understanding these interactions and how insects cope with the exposure to toxins can further our knowledge into how pest species develop resistance to insecticides and how to better protect beneficial insect species from exposure and harmful effects.

## 2. Exposure to Toxic Chemicals

### 2.1. Plant Defense Compounds

Many secondary metabolites produced by plants can function as anti-herbivory compounds, either as antifeedants or as toxins. Antifeedants act on the insect taste receptors, or sensilla, creating an unpleasant taste and deterring the insect herbivores from feeding [42,43,44,45]. Toxins, however, do not just deter insects, but cause some physiological harm to them. Some phytotoxins inhibit insect protease activity, interfering with the insect’s enzymatic breakdown of food and proper digestion [46,47]. Others target insect detoxification enzymes to reduce the insect’s ability to cope with toxin exposure [48,49]. Insects feeding on certain plants and plant-derived compounds can have reduced weight and inhibited development [49,50,51]. What follows is the “evolutionary arms race” between plants and insect herbivores, with plants evolving more chemical mechanisms to prevent damage by insect feeding and insect herbivores evolving new adaptations to cope with these chemical toxins and deterrents.

Some specialist insects will even preferentially feed on plants that produce toxic or bad-tasting secondary metabolites [52,53]. These compounds come in a variety of types. There are the cyanogenic glucosides, which are widely produced in many vascular plants, like sorghum, barley, and clover, and act as antifeedants [52]. Despite their ability to deter many herbivores from feeding, certain insects, such as the southern armyworm (*Spodoptera eridania*), will preferentially feed on plants that produce these cyanogenic glucosides [54]. Glucosinolates are another common group of defense compounds, often occurring in plants in the Order Brassicales (sometimes referred to as Capparales), including oilseed rape [52]. They are sulfur- or nitrogen-containing compounds, and have been shown to decrease aphid fecundity [55], though like the cyanogenic glucosides, some insects show a preference for glucosinolate-producing plants [56]. Many well-known plant phytotoxins are alkaloids, which are subdivided into three major classes: the true alkaloids, which includes nicotine; the pseudoalkaloids, such as caffeine; and the protoalkaloids, like mescaline. Other categories of toxic secondary metabolites include non-protein amino acids, phenolics, terpenes, and others [52]. Additionally, many of these secondary metabolites can form toxic products when metabolized by the herbivorous insects. For example, the glucosinolates, when hydrolyzed by myrosinase enzymes, will produce toxic isothiocyanates in the guts of herbivores [57].

An important plant signaling pathway to induce this defensive response against insect herbivores is the jasmonate pathway. Jasmonate, a hormone also involved in plant growth and development, can cause downstream response following stress, including wounding from insect feeding, drought, and fungal infection [58]. The downstream effects of this signaling pathway include upregulation of phytotoxins, such as nicotine and other alkaloids and glucosinolates to help deter herbivores [59].

Though these plant defense compounds primarily serve to prevent damage to the plant from herbivorous feeding, the nectar and pollen, which serve as rewards for pollinators, can also contain levels of anti-herbivory compounds. Some of these phytotoxins have been shown to negatively affect pollinator health and can potentially drive plant–pollinator evolution. Bumble bees exposed to lupanine, a quinolizidine alkaloid found in *Lupinus* spp. of plants, caused colonies to produce fewer and smaller males. Additionally, worker bees did not avoid pollen with lupanine, but fed it willingly to larvae [60]. In Europe, the invasive plant, *Rhododendron ponticum*, produced grayanotoxin I, which increased honey bee (*Apis mellifera*) mortality and deterred feeding of the mining bee, *Andrena carantonica*, though it seemed to have no effect on the bumble bee, *Bombus terrestris*. There have been several functions proposed for why plant toxins can be present in nectar, including deterring nectar robbers, attracting specialist pollinators, and preventing microbial growth [61,62]. Plants in the genus *Toxicoscordion* (formerly *Zigadenus*), for example, produce the alkaloid zygacine, which is toxic to several generalist pollinators, including *A. mellifera* and *Osmia lignaria*. The specialist bee, *Andrena astragali*, however, feeds exclusively on pollen from this genus [61,63,64]. Specialist feeders tend to be more efficient and are likely better adapted at pollinating certain flowers [65]. However, toxins in nectar and pollen may also be a remnant from past selection pressure or may have translocated to the nectar through the plant phloem [66]. These secondary metabolites in pollen and nectar may also impact pollinator behavior, in ways that can increase pollination activity and reproductive success of the plant [67]. Caffeine, for example, can stimulate honey bee memory and increase their fidelity for the plants with caffeine-containing nectar [68]. These interactions are complex as plant evolution is driven by the necessities of deterring harmful insect herbivores while attracting beneficial insect pollinators.

Levels of plant defensive compounds tend to be lower in nectar than in other plant tissues, which may negate some of the negative health effects of these compounds on pollinators [69,70]. In small concentrations, some of these compounds can even act as a reward for pollinators. Low doses of caffeine, a bitter tasting purine alkaloid, are naturally present in the nectar of plants in the *Coffea* and *Citrus* genera [53,62,68]. Honey bees that were fed small doses of caffeine showed both an increase in their olfactory memory and an increase in foraging and recruitment behaviors for flowers that provided caffeine in their nectar [68,71]. Doses of 0.01 M or higher, however, acted as a repellent and decreased honey bee acquisition of food rewards [72]. Likewise, concentrations of the norditerpene alkaloids that naturally occurred in the nectar of *Delphinium* spp. caused no noticeable deleterious effects on their bumble bee (*Bombus appositus*) pollinators. Only for concentrations 25× higher than those naturally occurring in nectar did the bumble bees show reduced mobility [69,70]. Looking at 32 *Nicotiana* species, as well, researchers found that outcrossing species, which are reliant on animal pollinators, had lower levels of nicotine in their leaves, flowers, and nectar than selfing species [73]. Though herbivores have been considered the primary drivers of plant defensive evolution, it seems likely that the need or lack of need for pollination also drives this. The reaction of pollinators exposed to these plant allelochemicals, either through gut symbionts, behavioral reactions, detoxification, or other methods, can help further our overall understanding of how insects cope with toxic xenobiotics.

### 2.2. Synthetic Pesticides

As well as naturally produced toxins, insects are also exposed to a myriad of synthetic agrochemicals. Many agricultural crop fields as well as non-agricultural areas, such as lawns and parks, are sprayed with herbicides for controlling weedy plants, which are generally not directly toxic to insects. They have, however, been shown to alter the gut bacterial communities (i.e., gut microbiome) of honey bees, reduce floral availability, and decrease plant diversity [13,74,75,76], and consequently they may have indirect effects on insects that forage on flowering weeds. Fungicides and acaricides are also considered to have low toxicity to most insects; however, they can have synergistic effects, increasing the toxicity of other pesticides [77]. The fungicide prochloraz, for example, increased the toxicity of the acaricides, tau-fluvalinate, coumaphos, and fenpyroximate in *A. mellifera* [78]. The fungicides triflumizole and propiconazole can greatly increase the potency of certain insecticides, such as thiacloprid and acetamiprid on *A. mellifera* [79]. The effects of insecticides on insects are often much more direct. The most commonly used insecticides target the nervous systems of insects, through the inhibition of acetylcholinesterase in the case of organophosphates (OPs) and carbamates, modification voltage-gated sodium channel in insect axons in the case of pyrethroids, and activation of nicotinic acetylcholine receptors in the case of neonicotinoids and sulfoximines [80]. Depending on the specificity of these insecticides, they can have low to high toxicity to nontarget organisms, as well as target pests [81,82,83,84].

Insects can be exposed to these pesticides through direct contact during application of foliar sprays, residual exposure, systemic activity from treated seeds or plant parts, and environmental contamination of water and soil [31,33,85,86,87]. Residues of neonicotinoids, such clothianidin, imidacloprid, and thiamethoxam, as well as other systemics, can be present in flowers, including wildflowers near the fields being sprayed or planted with neonicotinoid-treated seeds. These insecticides are often applied as seed treatments, but are then translocated through the plant tissues, where they can collect in the pollen and nectar [88,89]. Pollen containing these residues can then end up in a honey bee hive’s pollen stores and beeswax [33,90]. Surveys of pollinators, including native bees and honey bees, have found several pesticide residues both in the hive products and in the bees themselves. In one such survey, 70% of the bees sampled contained at least one pesticide and 48% had more than one [32]. A survey of North American apiaries found 121 pesticides and residues, the most common of which were acaricides (fluvalinate, coumaphos) and fungicides (chlorothalonil). The insecticides, aldicarb, carbaryl, imidacloprid, and captan, and the herbicide, pendimethalin, were also found frequently [32]. These insecticides can increase crop yield and allow for a higher production of food and cash crops to meet the demands of a rapidly increasing human population [91]. There is concern, however, with increasing pesticide use that beneficial insects like pollinators and natural enemies are being adversely affected and that the overuse of these pesticides is leading to a faster development of insecticide-resistant pests.

Modern insecticides generally have selective toxicity to insects while being less harmful to mammals and other vertebrate species. There are several reasons for this. Vertebrates and arthropods may have similar target sites, but key differences in enzyme and receptor structures make insects far more susceptible. Some toxins will bind to targets that are unique to insects and other arthropods, such as those involved in chitin synthesis and metamorphosis hormone receptors, and as such, are generally harmless to vertebrates. Finally, mammals and other vertebrates may have detoxification methods that render the toxins relatively harmless [92]. Due to the common presence of natural and synthetic toxins, however, insects have developed a myriad of ways to survive and adapt to them. These methods include target site insensitivity, such as ion channel mutation, sequestration of toxins [93], and cuticular protein mutation to reduce the absorption of toxins [94]. Insects have also developed means of actively degrading pesticides and plant phytotoxins, either through their mutualistic symbionts or by altering the regulation of their own enzymes. In this review, we discuss the role of insect microbial symbionts, particularly gut bacteria, in the detoxification of these environmental toxins. Understanding this can also help us combat pest species’ resistance to insecticides and better protect beneficial insects from toxin exposure.

## 3. Mechanisms of Insect Resistance to Xenobiotics

Insects that develop resistance to insecticides can be a persistent problem in agricultural and urban pest control. In many cases, resistance is quick to follow the release of a new insecticide. One example of this is the rapid development of cotton whitefly (*Bemisia tabaci*) resistance to the neonicotinoid, imidacloprid. Neonicotinoids began their release in 1991 as a safer alternative to organophosphate and carbamate insecticides, which had many insects already resistant to them [95,96]. Due to the unique mode of action of the neonicontinoids, which target the nicotinic acetylcholine receptors (nAChRs) of insects [97], they were highly effective against pests that were resistant to these older classes of insecticides [96,98]. In 1996, however, just 5 years after imidacloprid was made commercially available, *B. tabaci* was reported showing resistance to the insecticide [99]. Many other pest species began to show similar resistance to commonly used neonicotinoids and to other insecticides, as well. These species, such as the cotton whitefly, the green peach aphid, and the cotton-melon aphid, seem to be highly adaptable and able to quickly develop resistance [95].

Because of their exposure to phytotoxins, agrochemicals, and other toxins in their environment, many insects have evolved a variety of mechanisms to reduce the adverse effects of this exposure. This has resulted in resistant populations with distinct genetic differences to more susceptible populations. Resistance can be brought on by a change to a single gene (monogenic resistance), which usually occurs when a novel mutation provides greater resistance to an otherwise susceptible population. With high insecticide pressure, the resistant gene becomes more prevalent, and the entire population can gain resistance. More common, however, is resistance due to multiple gene changes (polygenic resistance). A subset of individuals in the insect population may be more resistant and so their full complement of genetic traits becomes dominant in the population through natural selection [100,101]. Phenotypically, this can result in behavioral changes, such as avoiding certain chemical cues. Many insects will avoid bitter tastes, such as the phytochemicals caffeine and quinine [102]. The tomato leafminer (*Tuta absoluta*), a moth pest of tomatoes, avoided ovipositing on plants that had been treated with azadirachtin, a secondary metabolite of the Neem tree (*Azadirachtin indica*) [103]. Not all toxins can be avoided by insects, however, and so they have also developed physiological changes, such as target-site mutations, sequestration of toxins, altered enzymatic activity, and microbial symbionts to deal with environmental toxins [104,105]. Mutations to the target sites of both natural and synthetic insecticides has conferred resistance for many species. *Tuta absoluta* moths with mutations in their ryanodine receptors (RyR), the target site of diamide insecticides, were more resistant to the diamides, chlorantraniprole and flubendiamide [106]. Other insects have evolved the ability to sequester toxins, usually from their host plants, and in turn use them against their own predators. The flea beetle (*Phyllotreta armoraciae*) sequesters plant glucosinolates out of the gut to prevent them from being hydrolyzed into isothiocyanates by myrosinase enzymes [57]. The western corn rootworm (*Diabrotica virgifera virgifera*) stores benzoazinoids, secondary metabolites of maize plants in their inactive form (protoxins). They are then able to activate the protoxins with their own activating enzymes to deter predation [107]. This sequestration can be concentration dependent, however. Colonies of the oleander aphid, *Aphis nerii*, will sequester cardenolide toxins from milkweed host plants for protection against ladybeetle predators. Milkweed species with higher levels of the cardenolides, however, such as *Asclepias curassavica*, limited the population growth of *A. nerii* [108].

Insects can also increase the expression of their detoxification enzymes, especially those within the cytochrome P450 monooxygenase (P450), glutathione-*S*-transferase (GST), and carboxylesterase families and superfamilies of enzymes for developing insecticide resistance and adapting to toxic environments [35,36,37,38,39,40,41]. These enzymes can be upregulated by several signaling pathways in insects, most notably the AhR/ARNT or xanthotoxin cascade, the CncC/Keap1 pathways, and the MAPK/CREB pathway. These cascades are triggered by the uptake of toxins and result in the movement of transcription factors to the nucleus of the insect’s cells. The transcription factors can then promote the transcription of genes for key detoxification enzymes [109,110]. The activity of these detoxification enzymes are classified into three phases: Phase I (functionalization), in which the enzymes render the toxin functionally unable to properly bind to target sites; Phase II (conjugation), in which the enzymes prepare the toxin or its metabolites for excretion; and Phase III (excretion), in which the toxin and it metabolites are moved out of the cells for excretion from the body [61,105,111].

Many microbial symbionts can aid in detoxification and improve the health and survival of their insect hosts following exposures to certain toxins. There are two primary mechanisms that these symbionts can use to do this. The first is with enzymes produced by the microbes, which can degrade toxic xenobiotics into less toxic or more easily secreted products [112,113,114]. An example of this is the organophosphate hydrolase (OPH) enzyme, which is found in several bacteria and fungi and can degrade organophosphate insecticides [112]. Bacterial symbionts have also been shown to affect the expression of host insect detoxification genes, and can influence the upregulation of insect GST and P450 enzymes in the presence of toxic xenobiotics [115,116]. These gut symbionts have the ability to rapidly confer resistance on their hosts, which makes the study of this microbial activity as necessary as studying the insect’s own physiological mechanisms.

## 4. Microbial Symbionts

Bacterial symbionts live in mutualistic relationships with insects and other animal species, providing benefits to the nutrition, digestion, and defenses of their hosts [117]. They can be categorized as either primary symbionts, which are essential for the host to properly live and function, or secondary symbionts. The primary symbionts tend to be maternally transmitted and ubiquitous in the host species, whereas secondary symbionts often have less fidelity to one host species. Secondary symbionts can still be highly common but are not necessarily omnipresent. Despite not being considered essential for the life of the host, secondary symbionts can provide many benefits and complex interactions, including increasing the host’s thermal tolerance and pathogen resistance [118,119]. Symbionts can have a tremendous impact on the lives of their hosts in a variety of ways, and they can influence the behavior, digestion, growth and development, immunity, and detoxification of their hosts [104,118,120,121,122].

The overall survival of bumble bees (*B. terrestris*) was higher with a typical gut biome than in bees treated with antibiotics [123,124]. Honey bee gut symbionts helped with weight gain in newly emerged adult bees, in part by upregulating the bee insulin/insulin-like signaling (IIS) pathway. They also increased the bees’ sensitivity to sucrose, which made bees with conventional gut biomes eat more and gain more weight than dysbiotic bees [125]. A similar effect was seen in mealworm beetles (*Tenebrio molitor*), which had reduced weight gain and premature pupation when raised aseptically or treated with antibiotics [126]. Symbionts can also provide some protection from parasitoids and pathogens. *Bombus terrestris* susceptibility to infection by the trypanosome parasite, *Crithidia bombi*, rose with higher concentrated doses of the antibiotic, oxytetracycline [123]. Honey bee gut symbionts can also play a key role in parasite and pathogen resistance. Asian honey bees (*Apis cerana*) are less susceptible to Israeli acute paralysis virus (IAPV) than western honey bees (*A. mellifera*), despite a similar innate immune response in both species. When treated with antibiotics, however, *A. cerana* showed higher mortality and infection rates due to IAPV, suggesting that their gut microbiome helps protect them from certain viral infections, like IAPV [127]. The bacterium, *Hamiltonella defensa*, an endosymbiont of aphids, is itself host to an APSE bacteriophage that produces toxins that can harm the eggs of parasitoid wasps, thus protecting the aphids [128]. Conversely, some parasitoid wasps depend on the gut microbial communities of their hosts for their proper development and growth. This was seen in *Leptopilina boulardi*, a parasitoid of the fruit fly, *Drosophila melanogaster*, which failed to develop in dysbiotic *D. melanogaster* that lacked a conventional gut microbiome [129]. Other symbiotic microbes can upregulate insect antimicrobial peptides (AMPs) and alter physiological conditions within the insect gut, lowering the pH and oxygen levels, all of which can reduce pathogen infection rates [130,131] In some cases, microbial symbionts can lower vector competence by reducing pathogen abundance. This was seen in the *Anopheles gambiae* mosquito, a vector of malaria, which had lower abundance and rates of the malaria causal agent, *Plasmodium falciparum*, when microbial symbiont abundance was higher [132,133]. These insect symbionts can influence insect health and resistance, as well as vector–pathogen interactions in ways that can impact human health.

Insects acquire their microbial symbionts in a variety of ways. Symbionts can be acquired via vertical transmission, from parent to offspring, but many are picked up from the environment [104]. Environmental bacteria can be acquired from the nest or larval habitat, from food sources, or from other insects [133,134]. Sociality can greatly influence transmission and consistency of microbial communities within insects. For termites, which depend on hindgut symbionts to aid in digestion, sociality can provide easy access to contaminated feces and facilitate the transfer of these vital symbionts [120]. In bees, the solitary species tend to have more varied gut communities, whereas the social bumble bees and honey bees have consistent core communities passed to them from their colony mates [134,135,136].

These environmentally acquired microbes can have high potential to confer resistance to the insects that acquire them. When the levels of certain toxins in an environment are altered, the relative abundance of the environmental microbial communities and gut microbial communities can change, as well. Heavy applications of insecticides, for example, have been shown to increase the environmental presence of bacteria that are able to degrade the specific insecticide [137,138,139]. If the bacteria are able to be acquired by insects in the field, they could then confer resistance to the insecticide on the insect [94,104]. Bacterial symbionts can excel at conferring resistance because they tend to have much more diverse genes and pathways for toxin degradation than the insects themselves, as well as a shorter lifecycle [53,104].

### 4.1. Bacterial Symbiont Degradation of Phytotoxins

Several insect herbivores are able to digest plant secondary metabolites that are considered toxic to other species, and some will even preferentially feed when these toxins are present. Though this field is still understudied, there are studies that support the role of microbial symbionts in the detoxification of these compounds. Gut microbial symbionts are most often implicated in detoxification, though there is evidence that salivary symbionts and plant-associated symbionts can play a role, as well. Many herbivorous insects can be pests of agricultural crops, forests, and stored grains, and most of the research into symbiont-based phytotoxin degradation has focused on these pest species and may provide future insights into improved control strategies (Table 1).

One such agricultural pest is the coffee berry borer (*Hypothenemus hampei*), which feeds exclusively on coffee beans, despite the high levels of caffeine. In other insects, caffeine, a toxic alkaloid, can cause deleterious health effects, including inhibition of phosphodiesterase activity and increasing cyclic AMP levels, which can cause paralysis. The coffee berry borer is able to feed on caffeine without these negative impacts in large part due to its gut microbiota and particularly due to the bacterium, *Pseudomonadales fulva*. This bacterium was able to degrade caffeine that passed through the beetles’ guts in vivo. In this case, the mechanism behind the degradation is believed to be through the bacterium’s monooxygenase activity, encoded by the *ndmA* gene, which is involved in the demethylation of caffeine [53].

Other studies into agricultural pests show the important role of symbiont-based phytotoxin degradation, even when the precise mechanism is not known. The cabbage stem flea beetle (*Psylliodes chrysocephala*), a pest of oilseed rape, has a gut microbial community capable of degrading isothiocyanates in vivo. Bacteria in the *Pantoea* genus seemed to play a particular role, and they were able to degrade isothiocyanates in vitro and restore degradation to beetles that had been fed antibiotics [140]. Another example is the camellia weevil (*Curculia chinensis*), which is a pest of *Camellia* spp. trees in China. *Camellia* trees are commonly used to make tea and oils and the seed-feeding by *C. chinensis* can cause damage. Beetles with whole gut microbiomes had higher survival and adult emergence rates than germ-free beetles or those with only one phylotype from the gut biome. This would suggest that not only can a single species aid in toxin resistance, but in some cases, the interactions between symbionts can play an important role [141]. The olive fly (*Bactrocera oleae*) also relies on its gut symbionts to aid in degradation. These flies are somewhat unique among the fruit flies in that their larvae develop in unripe olives, which can contain high levels of phenolic glycosides, such as oleuropein. Their gut microbial community, especially the obligate bacterial species, *Erwinia dacicola*, greatly improve *B. oleae* survival and development in these unripe olives, possibly from oleuropein degradation [142].

Similar trends have been seen in forest and stored grain pests. The mountain pine beetle (*Dendroctonus ponderosae*) feeds on live conifers, which can produce high levels of terpenes. These beetles possess a gut community with genes for terpene degradation, which may allow them to feed on the live trees [143]. However, more research is needed to see if there is a fitness cost in the beetles to feeding on terpenes with a dysbiotic microbiome. In Europe, the pine weevil (*Hylobius abietis*) also possesses a gut microbiota that can aid in terpenoid degradation. These gut microbes were able to degrade diterpenes that passed through the weevil guts in vivo and restore this degradation to antibiotic exposed weevils [144].

For stored grain pests, the mealworm beetle (*Tenebrio molitor*) showed fitness reduction and greater sensitivity to saligenin, and derivative of the alcoholic glucoside, salicin, produced by willow trees. Germ-free mealworms weighed less and pupated earlier than larvae with conventional gut biomes. These effects were amplified in germ-free larvae also exposed to saligenin, which also had higher mortality [126]. These effects would suggest that the mealworm microbiome plays a role in growth and development, as well as detoxification.

**Table 1:** Different types of symbionts found in insects and their role in detoxification of various xenobiotics.

Insect Species	Symbiont	Role of Symbiont	References
Coleoptera			
*Callosobruchus maculatus*	Gut bacteria	Dichlorvos degradation	[145]
*Curculio chinensis*	Gut community, *Acinetobacter* spp.	Triterpene saponin degradation	[141]
*Dendroctonus ponderosae*	Gut community	Genes for terpene degradation	[143]
*Hylobius abietis*	Gut community	Diterpene degradation	[144]
*Hypothenemus hampei*	*Pseudomonadales fulva*	Caffeine degradation	[53]
*Leptinotarsa decemlineata*	Salivary bacteria	Induction of salicylic-acid-based defense in host plant	[146]
*Psylliodes chrysocephala*	*Pantoea* spp.	Isothiocyanate degradation	[140]
*Tenebrio molitor*	Gut community	Saligenin degradation, weight gain	[126]
Diptera			
*Aedes aegypti*	Gut community	Lambda-cyhalothrin resistance	[147]
*Anopheles albimanus*	*Bacillus cereus*, other gut bacteria	Organophosphate degradation	[148]
*Bactrocera dorsalis*	*Citrobacter* sp. (CF-BD)	Organophosphate degradation	[149]
*Bactrocera oleae*	*Erwinia dacicola*	Possible oleuropein degradation	[142]
*Drosophila melanogaster*	Gut community	Nitro-reduced imidacloprid metabolism	[113]
*Rhagoletis pomonella*	*Pseudomonas melophthora*	Organophosphate degradation	[150]
Hemiptera			
*Nilaparvata lugens*	*Wolbachia, Arsenophonus*, *Acinetobacter*, and *Staphylococcus* spp.	Induction of planthopper degradation enzymes CncC pathway, imidacloprid degradation	[116,151]
*Riptortus pedestris*	*Burkholderia* spp.	Fenitrothion degradation	[138]
Hymenoptera			
*Apis mellifera*	Gut microbiome	Induction of bee degradation enzymes	[115]
*Bombus impatiens*	*Snodgrassella alvi*, *Lactobacillus bombicola*	Selenate degradation	[152]
*Nasonia vitripennis*	*Serratia marcescens*, *Pseudomonas pretegens*	Atrazine degradation	[137]
Lepidoptera			
*Lymantria dispar*	*Acinetobacter* sp. (R7-1), other gut microbes	Salicortin and tremulacin degradation	[153]
*Plutella xylostella*	*Enterococcus* sp, *Enterobacter* sp, and *Serratia* sp. *Enterobacter asburiae*, *Bacillus cereus*, and *Pantoea agglomerans*	Chlorpyrifos degradation Acephate degradation	[154,155]
*Spodoptera frugiperda*	*Laclercia adecarboxylata*, other gut bacteria	Degradation of chlorpyrifos ethyl, other insecticides	[156]

It is not just the gut microbial symbionts that have been implicated in insect resistance to plant defense, but also the salivary symbionts. Larvae of the Colorado potato beetle (*Leptinotarsa decemlineata*) secrete bacteria while feeding that help them overcome the plant’s defense response. The bacteria do this by inducing a salicylic acid (SA)-regulated defense response in the plant, which targets microbes [146]. The SA-regulated pathway has an inverse relationship with the jasmonate (JA)-regulated defense response pathway, which targets herbivores [58,146]. By inducing the SA-regulated pathway, the bacterial symbionts interfere with the plant’s JA-signaling and ability to defend against the insect herbivore. In this case, the microbes are not necessarily involved in degradation of toxins, in the prevention of phytotoxins against insects.

Finally, the microbe communities associated with plants can sometimes aid insect herbivores. An example of this was seen in the moth, *Lymantria dispar*, which obtains a large portion of its gut biome from its host plants. Because of its broad host range, *L. dispar* gut microbial communities can vary greatly based on location and common hosts. One study compared the detoxification ability of *L. dispar* larvae that were either germ-free, inoculated with bacteria from wild moths feeding primarily on oak trees, or inoculated with bacteria from aspen trees. They then exposed the larvae to phenolic glycosides, primarily salicortin and tremulacin, which are produced by aspen trees. They found that the bacteria from aspen trees were able to degrade the phenolic glycosides in vivo, but the bacteria from wild oak-feeding moths were not significantly better than the germ-free [153]. An *Acinetobacter* sp. (R7-1) was able to degrade salicortin and tremulacin in vitro, as well, though moth larvae inoculated with this bacteria species alone did not have the same benefits as the whole aspen bacterial community inoculation when exposed to phenolic glycosides, which would suggest again that in many cases the community interactions can play a key role [153]. All of these examples show that many insects and many important pest species benefit from their microbial symbionts to aid in their phytophagous activity.

### 4.2. Bacterial Symbiont Degradation of Synthetic Toxic Chemicals

Many insecticides have been developed to control populations of agricultural pests and disease vectors, though insects can gain resistance to these insecticides within a few years of their release [99]. In some cases, this can be due to their gut symbionts, as was seen in the bean bug (*Riptortus pedestris*). The bean bug nymphs acquire *Burkholderia* spp. of bacteria from contaminated soil, and some strains of *Burkholderia* are capable of degrading the organophosphate, fenitrothion, and conferring that resistance to their hosts. The fenitrothion-degrading strains of *Burkholderia* tend to be present at low levels in the environment; however, soil treated with fenitrothion caused the levels fenitrothion-degrading strains to increase. Likewise, the numbers of bean bugs containing the fenitrothion-degrading *Burkholderia* strains increased [138,157]. This conference of resistance can occur rapidly, as well, after only a few treatments of fenitrothion [157].

Several other agricultural pests gained insecticide resistance from their gut symbionts, particularly to organophosphate insecticides. Larvae of the *P. xylostella* moth contain microbial symbionts capable of degrading acephate. Three isolates in particular, *Enterobacter asburiae*, *Bacillus cereus*, and *Pantoea agglomerans*, were capable of this degradation and used acephate as a carbon and energy source [155]. Another study on *P. xylostella* found that three bacteria species, *Enterococcus* sp., *Enterobacter* sp., and *Serratia* sp., were capable of degrading the OP chlorpyrifos in vitro, though only the *Enterococcus* sp. improved *P. xylostella* resistance in vivo [154]. In comparing resistant and susceptible populations of fall armyworms (*Spodoptera frugiperda*) to chlorpyrifos ethyl, researchers found that resistant populations had higher proportions of the bacterium, *Laclercia adecarboxylata*, which may be capable of OP degradation [156]. Gut symbionts of the cowpea beetle (*Callosobruchus maculatus*) increased beetle survival after exposure to the organophosphate dichlorvos, but not to the phytochemicals in the essential oil made from the Gambian tea bush (*Lippia adoensis*), which acts as a natural insecticide [145]. All these studies show that many of these gut symbionts improve survival of agricultural pests to organophosphates.

Disease vectors, notably mosquitoes, can also have improved survival and resistance to insecticides due to their gut symbionts. *Anopheles albimanus*, a vector of malaria in South America and southern North America, had altered gut communities in populations that were resistant to fenitrothion compared to susceptible populations. More OP-degrading enzymes, carboxylesterases and phosphomonoesterases, were present in the bacterial metagenome of resistant mosquito guts, and the symbiont, *Bacillus cereus*, was only present in resistant mosquitos [148]. *Bacillus cereus* has been shown to be capable of OP degradation in previous studies [155], so it is a candidate for conferring resistance on *A. albimanus*. Another mosquito, *Aedes aegypti*, a vector of dengue, Zika, and chikungunya viruses, can develop resistance to the pyrethroid lambda-cyhalothrin from gut symbionts. Mosquitoes treated with antibiotics had increased sensitivity to lambda-cyhalothrin. Additionally, resistant mosquito populations had unique bacterial OTUs present and altered relative abundance, which may aid in the pyrethroid detoxification [147].

It is not just the pest insect species that can be exposed to pesticides and other toxic chemicals, however, but the beneficial species, as well. Pollinators, natural enemies, and decomposers can also encounter these toxins and develop resistance. The parasitoid wasp, *Nasonia vitripennis*, for example had greater sensitivity to the herbicide atrazine when reared in a sterile environment without exposure to typical gut microbiota. Wasps with conventional gut biomes which were then exposed to atrazine had alterations to their gut communities, which were then vertically transmitted to their offspring [137]. These changes increased resistance to atrazine within the population over a few generations, particularly due to the degradation activity of the symbionts *Serratia marcescens* and *Pseudomonas protegens*. Though *S. marcescens* was capable of atrazine degradation and improving *N. vitripennis* resistance, it can also act as an opportunistic pathogen and decrease wasp fitness if it reaches high densities in the gut [137]. Bumble bees (*Bombus impatiens*) also showed improved survival to the environmental toxins, in this case the metalloid selenate, when they had conventional gut microbiomes [152]. Selenate is a naturally occurring compound, but can be produced at higher concentrations through human activity, such as mining and coal combustion [158]. Bumble bee core bacteria, *Snodgrassella alvi* and *Lactobacillus bombicola*, were both able to grow in selenate-media and may be able to confer some resistance to the bumble bees [152].

Bacterial symbionts have two primary mechanisms for improving host survival after toxic chemical exposure: the degradation of the compound by the bacterium’s own enzymes and the upregulation of host detoxification genes. For the first mechanism, one of the earliest observed cases of symbiont-based resistance was in the apple maggot (*Rhagoletis pomonella*) and its symbiont, *Pseudomonas melophthora*, which was capable of OP degradation. Researchers thought this was likely due to the esterase activity of *P. melophthora* [150]. Later, other bacteria and fungi were also found to be able to degrade organophosphates due to their organophosphate hydrolase (OPH) enzymes, which hydrolyzed the P-O-alkyl and P-O-aryl bond of OPs and resulted in less toxic products [112]. Soil bacteria capable of degrading cypermethrin had increased esterase and laccase activity [114]. In insect symbionts, the bacterium *Citrobacter* sp. (CF-BD) contains genes for phosphatase hydrolases, which can also degrade OPs. CF-BD can then improve the survival of its host, the fruit fly *Bactrocera dorsalis*, when exposed to OPs like trichlorfon [149]. Though insects possess their own detoxification enzymes, bacteria can sometimes metabolize compounds using different pathways than insects. This was seen in *Drosophila melanogaster* fruit flies and their symbionts. The flies have a P450 enzyme, CYP6G1, which can metabolize the neonicotinoid, imidacloprid, using the oxidative pathway but not the nitro-reduced pathway. However, imidacloprid products from nitro-reduction can still be found in *D. melanogaster* with conventional microbiomes, but not in germ-free fly larvae. The microbial symbionts seems to be primarily responsible for the nitro-reduced metabolism of imidacloprid [113].

Different strains of bacteria can vary in their effectiveness for toxin degradation, however, using different enzymatic pathways for toxin degradation, which can lead to the production of toxic or less toxic metabolites. The broad-spectrum organophosphate insecticide, chlorpyrifos (CP), for example, can be broken down into the metabolites chlorpyrifos oxon (CPO), which is also highly toxic, and the less toxic 3,5,6-trichloro-2-pyridinol (TCP). In the model fruit fly species *Drosophila melanogaster*, the gut bacteria *Lactobacillus plantarum* will degrade CP into the more toxic CPO metabolite. Researchers found that *D. melanogaster* treated with antibiotics survived better than untreated flies when both groups were exposed to CP. Alternatively, when flies were treated with the probiotic *Lactobacillus rhamnosus* GG strain, which binds but does not metabolize CP, their survival increased when exposed to CP. Bacterial strains of *Lactobacillus* seemed to affect how the CP was metabolized and how well the host fruit flies were able to survive the toxins [159].

As well as their own enzymatic activity, microbes can influence host gene expression. The bacterial symbionts of the brown planthopper (*Nilaparvata lugens*) increased GST and P450 activity of the planthopper and improved its survival following exposure to imidacloprid, chlorpyrifos, and clothianidin. The genera *Wolbachia*, *Arsenophonus*, *Acinetobacter*, and *Staphylococcus*, in particular, were associated with increased activity of the planthopper detoxification enzymes [116]. Honey bee gut symbionts also increased the P450 activity of the bees and improved survival after thiacloprid or *tau*-fluvalinate exposure. Cultures of bee gut microbes were unable to metabolize thiacloprid in vitro, however, which would suggest that induction of host detoxification plays more of a role in the improved host survival, rather than bacterial enzymatic degradation [115].

The enzymatic activity of these microbes, as well as conferring resistance on host, offers potential for bioremediation of areas with accumulated toxic compounds. How these bacterial symbionts interact with their hosts can also offer new insights into pest control tactics and new ways to mitigate harm to beneficial insects.

## 5. Conclusions

Insects are exposed to a variety of toxic xenobiotics, both naturally occurring and synthetically produced. They can be exposed to these toxins through plant feeding, contaminated soils, exposure to foliar sprays, and several other routes of exposure. Because of this, insects have had to develop mechanisms to cope with toxin exposure, from enzymatic degradation to sequestration and rapid secretion. The symbiosis between certain host insects and their gut bacterial communities can play a vital role in insect resistance to toxins. As such, they can be used to gain new insights into host–symbiont relationships, as well as having practical applications in pest control and protection of beneficial insects.

In the field of pest management and disease vector control, understanding the mechanisms of insect detoxification and therefore the ways in which insects become resistant to insecticides is vital in order to further protect human health, food crops, and the agricultural economy. Targeting the gut microbial communities of pest species could be a way to reduce their resistance to insecticides or impact their health in other ways [160]. It is also important, however, to consider the health of ecosystems and to protect the populations of beneficial insects, such as pollinators and decomposers. The integrated pest management (IPM) or integrated pest and pollinator management (IPPM) framework offers a practical solution to both improve pollinator health [161,162] and decrease the occurrence of pesticide tolerant insect pests. The strategies of IPM include the use of biological and mechanical insect controls, rather than relying solely on chemical methods. Because the over-spraying of insecticides has adversely affected pollinator populations and increased the rate at which insects develop resistance, IPM provides the opportunity to make educated and informed decisions about pest control. Along with reducing pollinator exposure to pesticides, the use of probiotics in honey bee hives has been suggested to reinoculated bees with beneficial bacteria following antibiotic exposure. Bacterial symbionts could also be engineered to better protect beneficial insects from both insecticide exposure and pathogens.

It is worth noting that when studying the relationship among insects, their gut microbiomes, and resistance to toxins, the majority of the research has been done on bacterial symbionts. The role of fungal and viral interactions with bacteria and insect hosts is an area that could benefit from more research. Viruses like the APSE bacteriophage, which can reduce parasitism rates for their insect hosts, show a positive impact that viruses can have on insect health. The impacts that viruses, fungi, or other microbes could have on detoxification and conference of resistance to host organisms is still understudied, however. The interactions among all these microorganisms and the macroorganisms that they inhabit can be complicated but can provide new insights and practical applications to better manage insect populations.

## Data Availability

Data available upon request.
